# Loop diuretics in heart failure: The objective markers to guide the therapy are needed

**DOI:** 10.1002/ehf2.14920

**Published:** 2024-06-24

**Authors:** Jan Biegus, Robert Zymliński, Piotr Ponikowski

**Affiliations:** ^1^ Institute of Heart Diseases Wroclaw Medical University Wroclaw Poland

Congestion is a major clinical sign of acute heart failure (AHF), and loop diuretics remain the first‐line treatment option for decongestion in the acute phase of the disease.^1^ Even though diuretics facilitate water and sodium excretion and, therefore, enable more effective decongestion, we need to keep in mind that there are several drawbacks related to their use.

First of all, due to their mode of action, diuretics remove water through sodium‐related mechanisms, which in the long run may lead to sodium depletion. Although it has never been demonstrated, at least part of the ominous signs of heart failure—hyponatremia and hypochloremia—may be related to chronic exposure to loop diuretics.^2^, ^3^, ^4^ On the other hand, because sodium is needed for an adequate diuretic response (to loop diuretics), and at the same time, loop diuretics remove sodium from the body, a sort of vicious circle is created that hinders the diuretic response in the long run. In this mechanism (sodium depletion), diuretics probably indirectly activate the renin‐angiotensin‐aldosterone (RAA) system, which is meant to keep sodium homeostasis in our body. In addition, diuretics were shown to directly excite the RAA system by blocking the macula densa and thus promoting renin release. Furthermore, the excessive (diuretic mediated) sodium and water loss is counterbalanced with excessive thirst, and therefore, another vicious circle may be created.^5^

Some premises exist that the diuretic effect of most drugs (especially diuretics and SGLT‐2) gets blunted with time once some adaptive mechanisms come into play.^6^, ^7^ This is probably why the more advanced HF patients (patients who take diuretics for longer periods of time) need higher doses of the drugs.^8^ The diuretic response is also highly related to the neurohormonal drive, which can also identify patients with more disrupted pathophysiology and worse outcomes.^9^, ^10^, ^11^ Several studies have demonstrated the association between the dose of diuretics and the outcomes, but this has never been demonstrated in a casual‐effect manner as most of the data come from retrospective analyses or registries.^12^, ^13^ Unfortunately, at the moment, we lack objective measures that allow us to adjust the loop diuretic doses in HF patients. Thus, all dose modifications (up‐ and down‐titrations) are made based on the physician's subjective decisions, which can also lead to some bias.

Finally, there is no evidence that supports the notion that diuretics improve outcomes in HF. Thus, recently, novel hypotheses about different ways to decongest AHF patients have shown effectiveness, such as using SGLT‐2 inhibitors, ARNI, or optimizing guideline‐directed medical therapy (GDMT), as demonstrated in the STRONG‐HF trial.^14^, ^15^, ^16^ Taking into consideration all those aspects, it is not surprising that there is a growing interest in the de‐escalation of diuretics in HF patients.

On the other hand, several studies have demonstrated that congestion and residual congestion (in particular) are associated with worse outcomes. But again, the direct causality cannot be derived from those observations as most of the data come from retrospective or observational studies. It is, therefore, very likely that it is much more difficult to decongest sicker patients, and this creates an association between residual congestion and unfavourable outcomes. Diuretic responsiveness and diuretic efficiency are net results of the advancement of the disease and adaptive mechanisms that blunt the responsiveness in the long run rather than the drug characteristics themselves.

Both of these aspects have been studied in a recent paper published in ESC Heart Failure.^17^ Croset et al. have demonstrated that the down‐titration of loop diuretics at discharge from the hospital is safe in some patients.^17^ The authors have retrospectively analysed the cohort of mostly (80%) heart failure with preserved ejection fraction patients who were hospitalized for AHF in a single centre. The doses of loop diuretics at admission and discharge were recorded and compared. This has led to the identification of patients with two different dose profiles: down titration and stable/up‐titration of diuretics during hospitalization. Importantly, the authors have also analysed both profiles in comparison with congestion status, defined by congestion score and some congestion biomarkers.

There are several important takes from the paper. Firstly, the down titration of loop diuretics was not associated with an increased risk of the composite endpoint (time to all‐cause death and/or HF rehospitalization) in the whole population. This is important and clinically relevant information as some clinicians may be very reluctant to down‐titrate diuretic doses only due to the fear of potential side effects, namely, fluid retention and subsequent decompensation. However, as demonstrated by the authors, this was not the case in the retrospectively analysed cohort.

Secondly, approximately one out of eight patients had the loop diuretic down‐titrated during the hospitalization. Taking into account the well‐described pharmacological inertia, the clinical circumstances (patient hospitalized due to some degree of congestion), and ‘natural’ reluctance to optimize the HF treatment during hospitalization, the reported number is quite high. We must keep in mind that hospitalization is a great (but usually unused or misused) opportunity to modify and optimize the pharmacotherapy of HF patients.^18^, ^19^

Thirdly, it is important to stress that the down‐titration group started from high loop diuretic doses of a median of 100 mg and was discharged at a median dose of 50 mg. On the other hand, the increase/stable dose group had an up‐titration of loop diuretics of 40 mg and was discharged at a median dose of 80 mg. The differences in discharge doses and the changes made during hospitalization seem significant from a clinical perspective. But again, they were not related to worse outcomes.

Lastly, one important caveat must be emphasized. The down‐titration of diuretics was safe only in the subgroup of patients with lower clinical/laboratory signs of congestion. This is crucial information and probably a key to understanding the presented results. The down‐titration was associated with a higher risk of the study endpoints in a subgroup of patients with residual congestion, higher BNP (>985 pg/mL), and/or elevated Ca125 (>120 U/mL) at admission. This confirms the need for individualized therapy in some aspects of HF management, which may be a key to long‐term success.

Although the paper presents a clear clinical message, it is important to consider its limitations. It is a single‐centre retrospective analysis with all the drawbacks associated with this type of analysis. The study was also underpowered to detect small differences in study outcomes between both groups, and longer follow‐up might reveal some additional information.

To conclude, the study by Croset et al. has demonstrated the real clinical unmet need for objective markers to guide the loop diuretic dosing (both down‐ and up‐titration) in HF patients. The use of natriuretic‐guided decongestion is the first step for more individualized therapy, but there is still a long way to go. Moreover, the prognostic information of spot urine sodium is different at admission and discharge, which makes this even more complicated.^20^ The study has also shown that in a subpopulation of AHF patients, the down‐titration of diuretics was safe, as it was not associated with clinical deterioration. However, a prospective randomized study would be needed to provide definitive results.

## References

[1] McDonagh TA , Metra M , Adamo M , *et al*. 2021 ESC guidelines for the diagnosis and treatment of acute and chronic heart failure. Eur Heart J 2021;42:3599‐3726. doi:10.1093/eurheartj/ehab368 34447992

[2] Xia YM , Wang S , Wu WD , Liang JF . Association between serum sodium level trajectories and survival in patients with heart failure. ESC Heart Fail 2023;10:255‐263. doi:10.1002/ehf2.14187 36193558 PMC9871655

[3] Llacer P , Croset F , Nunez J , Campos J , Fernández C , Fabregate M , *et al*. Prognostic significance of plasma chloride in elderly patients hospitalized for acute heart failure. ESC Heart Fail 2023;10:2637‐2647. doi:10.1002/ehf2.14434 37349910 PMC10375113

[4] Seo M , Watanabe T , Yamada T , Yano M , Hayashi T , Nakagawa A , *et al*. Prognostic significance of serum chloride level in heart failure patients with preserved ejection fraction. ESC Heart Fail 2022;9:1444‐1453. doi:10.1002/ehf2.13840 35137570 PMC8934985

[5] van der Wal MHL , Jaarsma T , Jenneboer LC , Linssen GCM . Thirst in stable heart failure patients; time to reconsider fluid restriction and prescribed diuretics. ESC Heart Failure 2022;9:2181‐2188. doi:10.1002/ehf2.13960 35546481 PMC9288740

[6] Biegus J , Zymlinski R , Testani J , Fudim M , Cox ZL , Guzik M , *et al*. The blunted loop diuretic response in acute heart failure is driven by reduced tubular responsiveness rather than insufficient tubular delivery. The role of furosemide urine excretion on diuretic and natriuretic response in acute heart failure. Eur J Heart Fail 2023;25:1323‐1333. doi:10.1002/ejhf.2852 37042083

[7] Marton A , Saffari SE , Rauh M , Sun RN , Nagel AM , Linz P , *et al*. Water conservation overrides osmotic diuresis during SGLT2 inhibition in patients with heart failure. J Am Coll Cardiol 2024;83:1386‐1398. doi:10.1016/j.jacc.2024.02.020 38599715

[8] Tomasoni D , Vishram‐Nielsen JKK , Pagnesi M , Adamo M , Lombardi CM , Gustafsson F , *et al*. Advanced heart failure: guideline‐directed medical therapy, diuretics, inotropes, and palliative care. ESC Heart Fail 2022;9:1507‐1523. doi:10.1002/ehf2.13859 35352499 PMC9065830

[9] Matsumoto S , Nakamura N , Konishi M , Shibata A , Kida K , Ishii S , *et al*. Neuroendocrine hormone status and diuretic response to atrial natriuretic peptide in patients with acute heart failure. ESC Heart Fail 2022;9:4077‐4087. doi:10.1002/ehf2.14083 36043451 PMC9773655

[10] Zymlinski R , Sierpinski R , Metra M , Cotter G , Sokolski M , Siwołowski P , *et al*. Elevated plasma endothelin‐1 is related to low natriuresis, clinical signs of congestion, and poor outcome in acute heart failure. ESC Heart Fail 2020;7:3536‐3544. doi:10.1002/ehf2.13064 33063475 PMC7755016

[11] Biegus J , Nawrocka‐Millward S , Zymlinski R , Fudim M , Testani J , Marciniak D , *et al*. Distinct renin/aldosterone activity profiles correlate with renal function, natriuretic response, decongestive ability and prognosis in acute heart failure. Int J Cardiol 2021;345:54‐60. doi:10.1016/j.ijcard.2021.10.149 34728260

[12] Baudry G , Coutance G , Dorent R , Bauer F , Blanchart K , Boignard A , *et al*. Diuretic dose is a strong prognostic factor in ambulatory patients awaiting heart transplantation. ESC Heart Fail 2023;10:2843‐2852. doi:10.1002/ehf2.14467 37408178 PMC10567662

[13] Seko Y , Kato T , Morimoto T , Yaku H , Inuzuka Y , Tamaki Y , *et al*. Association between changes in loop diuretic dose and outcomes in acute heart failure. ESC Heart Fail 2023;10:1757‐1770. doi:10.1002/ehf2.14338 36858382 PMC10192247

[14] Biegus J , Voors AA , Collins SP , Kosiborod MN , Teerlink JR , Angermann CE , *et al*. Impact of empagliflozin on decongestion in acute heart failure: The EMPULSE trial. Eur Heart J 2023;44:41‐50. doi:10.1093/eurheartj/ehac530 36254693 PMC9805406

[15] Mebazaa A , Davison BA , Biegus J , Edwards C , Murtagh G , Varounis C , *et al*. Reduced congestion and improved response to a fluid/sodium challenge in chronic heart failure patients after initiation of sacubitril/valsartan: The NATRIUM‐HF study. Eur J Heart Fail 2024;n/a. doi:10.1002/ejhf.3265 38721803

[16] Bohm M , Assmus B , Anker SD , Asselbergs FW , Brachmann J , Brett M‐E , *et al*. Less loop diuretic use in patients on sacubitril/valsartan undergoing remote pulmonary artery pressure monitoring. ESC Heart Fail 2022;9:155‐163. doi:10.1002/ehf2.13665 34738340 PMC8787966

[17] Croset F , Llacer P , Nunez J , Campos J , García M , Pérez A , *et al*. Loop diuretic down‐titration at discharge in patients hospitalized for acute heart failure. ESC Heart Fail 2024;11:1739‐1747. doi:10.1002/ehf2.14749 38454739 PMC11098660

[18] Biegus J , Moayedi Y , Saldarriaga C , Ponikowski P . Getting ahead of the game: in‐hospital initiation of HFrEF therapies. Eur Heart J Suppl 2022;24:L38‐L44. doi:10.1093/eurheartjsupp/suac120 36545227 PMC9762886

[19] Palin V , Drozd M , Garland E , Malik A , Straw S , McGinlay M , *et al*. Reduction of heart failure guideline‐directed medication during hospitalization: prevalence, risk factors, and outcomes. ESC Heart Failure 2022;9:3298‐3307. doi:10.1002/ehf2.14051 35796239 PMC9715809

[20] Biegus J , Zymlinski R , Fudim M , Testani J , Sokolski M , Marciniak D , *et al*. Spot urine sodium in acute heart failure: Differences in prognostic value on admission and discharge. ESC Heart Fail 2021;8:2597‐2602. doi:10.1002/ehf2.13372 33932273 PMC8318409

